# Interrater Reliability of National Institutes of Health Traumatic Brain Injury Imaging Common Data Elements for Brain Magnetic Resonance Imaging in Mild Traumatic Brain Injury

**DOI:** 10.1089/neu.2021.0138

**Published:** 2021-10-12

**Authors:** Sandra P. Rincon, Pratik Mukherjee, Harvey S. Levin, Nancy R. Temkin, Christine L. Mac Donald, Daniel M. Krainak, Xiaoying Sun, Sonia Jain, Sabrina R. Taylor, Amy J. Markowitz, Allison Kumar, Geoffrey T. Manley, Esther L. Yuh

**Affiliations:** ^1^ Department of Radiology, Massachusetts General Hospital, Boston, Massachusetts, USA.; ^2^ Department of Radiology and Biomedical Imaging, University of California, San Francisco, California, USA.; ^3^ Brain and Spinal Injury Center at Zuckerberg San Francisco General Hospital, San Francisco, California, USA.; ^4^ Department of Physical Medicine and Rehabilitation, Baylor College of Medicine, Houston, Texas, USA.; ^5^ Department of Neurological Surgery, University of Washington, Seattle, Washington, USA.; ^6^ U.S. Food and Drug Administration (FDA), Silver Spring, Maryland, USA.; ^7^ Biostatistics Research Center, Herbert Wertheim School of Public Health and Longevity Science, University of California, San Diego, La Jolla, California, USA.; ^8^ Department of Neurological Surgery, University of California, San Francisco, California, USA.; ^9^ Arina Consulting, San Francisco, California, USA.

**Keywords:** Common Data Elements, FDA Medical Device Development Tool, imaging, interrater reliability, MRI, radiology

## Abstract

The National Institutes of Health/National Institute of Neurological Disorders and Stroke (NIH-NINDS) Traumatic Brain Injury (TBI) Imaging Common Data Elements (CDEs) are standardized definitions for pathological intracranial lesions based on their appearance on neuroimaging studies. The NIH-NINDS TBI Imaging CDEs were designed to be as consistent as possible with the U.S. Food and Drug Administration (FDA) definition of biomarkers as “an indicator of normal biological processes, pathogenic processes, or biological responses to an exposure or intervention.” However, the FDA qualification process for biomarkers requires proof of reliable biomarker test measurements. We determined the interrater reliability of TBI Imaging CDEs on subacute brain magnetic resonance imaging (MRI) performed on 517 mild TBI patients presenting to 11 U.S. level 1 trauma centers. Three U.S. board-certified neuroradiologists independently evaluated brain MRI performed 2 weeks post-injury for the following CDEs: traumatic axonal injury (TAI), diffuse axonal injury (DAI), and brain contusion. We found very high interrater agreement for brain contusion, with prevalence- and bias-adjusted kappa (PABAK) values for pairs of readers from 0.92 [95% confidence interval, 0.88–0.95] to 0.94 [0.90–0.96]. We found intermediate agreement for TAI and DAI, with PABAK values of 0.74–0.78 [0.70–0.82]. The near-perfect agreement for subacute brain contusion is likely attributable to the high conspicuity and distinctive appearance of these lesions on T1-weighted images. Interrater agreement for TAI and DAI was lower, because signal void in small vascular structures, and artifactual foci of signal void, can be difficult to distinguish from the punctate round or linear areas of slight hemorrhage that are a common hallmark of TAI/DAI on MRI.

## Introduction

Nearly 5 million patients are evaluated annually in U.S. emergency departments (EDs) for acute traumatic brain injury (TBI), 95% of which is mild TBI (mTBI; Glasgow Coma Scale [GCS] 13–15).^1^ Although post-concussive symptoms and impaired cognition often resolve within 6 months, a subgroup of mTBI patients experience persistent sequelae.^2–5^ Motivated by the realization that TBI classification schemes based primarily on the GCS score^6^ do not account for the diversity in TBI outcomes,^7,8^ the National Institute of Neurological Disorders and Stroke (NINDS) of the National Institutes of Health (NIH) in 2010 established the NIH-NINDS TBI Common Data Elements (CDEs), consisting of standardized data-collection protocols, clinical assessments, and outcome measures in TBI.^9,10^ The emphasis was on promoting uniformity and reproducibility of TBI classification and outcome assessment, in order to improve outcome prediction and increase the power of therapeutic multi-center clinical trials to uncover treatment effects through informed patient selection.

The NIH-NINDS TBI *Imaging* CDEs are a major component of the NIH-NINDS TBI CDEs. They consist of detailed definitions of pathological intracranial lesions and their appearance on neuroimaging studies. The TBI Imaging CDEs were designed to be consistent with the U.S. Food and Drug Administration (FDA) definition of biomarkers as a “defined characteristic that is measured as an indicator of normal biological processes, pathogenic processes, or biological responses to an exposure or intervention…”^11^ Two CDEs, brain contusion and diffuse axonal injury (DAI) on brain magnetic resonance imaging (MRI), have been suggested to have prognostic significance in mTBI.^12^ One of these, brain contusion on MRI, identified by a board-certified neuroradiologist, was recently qualified by the FDA Medical Device Development Tool (MDDT) program as a prognostic enrichment tool for mTBI clinical trials.^13^ An essential part of the FDA MDDT qualification process was to demonstrate that CDEs, including brain contusion, can be determined reliably. Here, we report the results of that effort. To our knowledge, this is the first report of the interrater reliability of NIH-NINDS TBI Imaging CDEs on brain MRI assessed by neuroradiologists.

## Methods

### Study population

The Transforming Research and Clinical Knowledge in Traumatic Brain Injury (TRACK-TBI) study is a prospective, longitudinal, observational study of acute TBI. The inclusion criterion was presentation to a participating level 1 trauma center within 24 h of injury with clinical indication for head computed tomography (CT) under American College of Emergency Medicine/Centers for Disease Control and Prevention criteria. Participating centers included Ben Taub General Hospital (Houston, TX), Massachusetts General Hospital (Boston, MA), Zuckerberg San Francisco General (San Francisco, CA), University of Cincinnati Medical Center (Cincinnati, OH), R. Adams Cowley Shock Trauma Center (Baltimore, MD), Ryder Trauma Center (Miami, FL), University of Pittsburgh Medical Center (Pittsburgh, PA), Seton Medical Center (Austin, TX), Parkland Memorial Hospital (Dallas, TX), Harborview Medical Center (Seattle, WA), and Virginia Commonwealth University Medical Center (Richmond, VA).

Exclusion criteria included pregnancy, incarceration, non-survivable physical trauma, and pre-existing medical/neuropsychiatric conditions that could interfere with outcome assessments. Patients or their legal representatives gave written informed consent. Participating centers' institutional review boards approved all study protocols. The present study is limited to the 517 TRACK-TBI patients 18–65 years of age at time of enrollment (February 26, 2014 to May 4, 2016), with GCS 13–15 upon ED arrival and who completed 2-week brain MRI.

### Brain magnetic resonance imaging and interpretation by three US board-certified neuroradiologists

Brain MRI, including T1, T2, T2-weighted fluid-attenuated inversion recovery (FLAIR), and T2*-weighted series, was performed at 7–18 days post-injury. All sites used a standardized MRI protocol^14^ across General Electric (General Electric Healthcare, Waukesha, WI), Siemens (Siemens Healthineers, Erlangen, Germany), and Phillips (Philips Healthcare, Best, The Netherlands) MRI platforms. MRI exams were deidentified and uploaded to a central repository.

Three neuroradiologists with American Board of Radiology certification and Certificate of Added Qualification in Neuroradiology independently reviewed each MRI exam. They had 17, 16, and 10 years of attending neuroradiologist experience and had completed neuroradiology fellowship training at different institutions (Massachusetts General Hospital [Boston, MA]; University of California, San Francisco [San Francisco, CA]; and Mallinckrodt Institute of Radiology, Washington University [St. Louis, MO]).

Each neuroradiologist reviewed the NIH-NINDS TBI Imaging CDE definitions before the study.^9,10^ Readers used FDA-cleared OsiriX software (Pixmeo, Geneva, Switzerland) to view MRI exams on an iMac with Retina display (Apple, Cupertino, CA). Readers were asked to annotate each lesion on at least one image by drawing an arrow pointing to the lesion or by encircling the lesion with a circle/oval. Readers were asked to annotate all brain contusions, though it was decided that enumeration of contusions would not be emphasized in this study; this was felt by all readers to be ambiguous, given that nearly contiguous contusions could be labeled as a single lesion or multiple lesions.

For traumatic axonal injury (TAI)/diffuse axonal injury (DAI), readers annotated as many lesions as possible, up to (when present) at least four lesions and including (when present) lesions in at least two lobes of the brain. Readers were free to choose any series or plane on which to annotate a lesion, but generally did so on an image of a series on which they felt the lesion was clearly demonstrated. For each annotated lesion, the reader entered CDE information using a pop-up dialog box created using an OsiriX TBI Imaging CDE plug-in module. Each neuroradiologist evaluated all 517 MRI exams without feedback regarding agreement with other readers. Readers had no access to clinical information except age/sex, and no access to previous head CT or other imaging exams.

### Brain contusion

In the NIH-NINDS TBI Imaging CDEs, brain contusion is defined as “a focal area of brain parenchymal disruption due to acute mechanical deformation. Contusions typically occur in the cortex and may extend into subcortical region… [F]or purposes of categorization, contusions are differentiated from ‘intracerebral hematomas' by containing a *mixture* of hemorrhagic and non-hemorrhagic tissue, or by having no grossly visible hemorrhage (‘bland contusion’), while an ‘intracerebral hematoma’ is predominantly a uniform collection of blood alone. The term ‘contusion’ should not be used for hemorrhagic lesions which fit better in other categories, such as small hemorrhages associated with the pattern of diffuse axonal injury, lesions which in context are more likely to represent infarction or other primary vascular lesion, or isolated subarachnoid hemorrhage.”^10^

### Traumatic axonal injury and diffuse axonal injury

In the NIH-NINDS TBI Imaging CDEs, TAI and DAI are defined as “a pattern consistent with scattered, small hemorrhagic and/or non-hemorrhagic lesions which have been shown historically to correlate with pathologic findings of relatively widespread injury to white matter axons, typically due to mechanical strain related to rotational acceleration/deceleration forces.” The definition further states that “‘diffuse axonal injury’ refers to a widespread distribution of lesions, including the subcortical white matter in more than one lobe or hemisphere, along with lesions in the corpus callosum, and may include the dorsomedial midbrain and other brainstem and cerebellar regions. ‘Traumatic axonal injury’ refers to similar multiple, scattered, small hemorrhagic and/or non-hemorrhagic lesions in a more confined white matter distribution… DAI includes more than three separate foci of signal abnormality, and TAI is 1-3 foci of signal abnormality.”^10^

### Statistical analysis

Interrater agreement for each CDE, brain contusion, and TAI/DAI was determined using Cohen's kappa (with 95% confidence intervals [CIs]) for each pair of neuroradiologists. Interrater agreement for each CDE was determined at a per-exam (i.e., per-patient) level, not at the individual lesion level. CDEs such as extra-axial collections present on initial CT, but that had often resolved or were barely perceptible by the time of 2-week MRI, were not analyzed. We also calculated prevalence- and bias-adjusted kappa values (PABAK; with 95% CIs) to account for low prevalence of CDE lesions in our mTBI population. Finally, to address previously raised limitations of Cohen's kappa,^15^ we determined overall percent agreement (OPA), positive percent agreement (PPA), and negative percent agreement (NPA) for brain contusion and for DAI, including 95% CIs using a bootstrap procedure (1000 resampled sets, each with 517 randomly selected datapoints from the original list of 517 data points, performed separately for each pair-wise OPA, PPA, and NPA).

### Consensus review

After readers had interpreted all 517 cases, they performed a consensus review to better understand sources of discrepancies. Readers discussed their own and others' annotations and, by majority vote, formed a final decision regarding the presence of at least one brain contusion on each exam and presence of TAI or DAI on each exam. Exams for which the initial interpretation for presence of a CDE was not unanimous, but were ultimately determined not to contain the CDE after a majority vote, were attributed to a discordance in the interpretation of perceived finding(s). Exams for which the initial interpretation for the presence of a CDE was not unanimous, but were determined to contain the CDE after a majority vote, could have been attributable to a discordance in the interpretation of perceived finding(s) and/or initial failure to perceive (detect) an abnormality (“I didn't see it”). For TAI, a consensus decision on the number of lesions was also recorded.

## Results

**FIG. 1.**
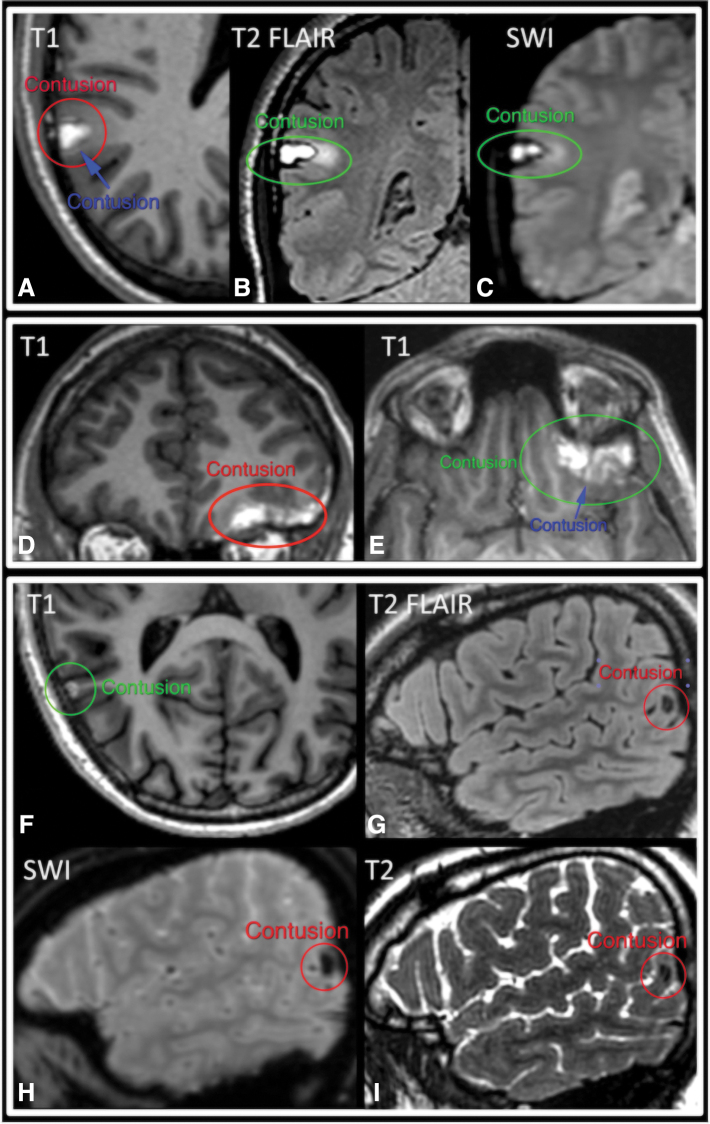
Brain contusion. Two examples of unanimous (**A–C, D,E**) and one example of partial agreement (**F–I**) across three neuroradiologists. “Partial agreement” refers to an MRI exam for which the three readers' initial interpretations regarding presence versus absence of a contusion on the exam were not unanimous. Annotations from independent readers are shown in different colors (red, green, and blue) superimposed on the same image for demonstration purposes. Brain contusions generally demonstrate high T1 signal in the 2-week subacute time frame (**A, D–F**), and they are typically very conspicuous against the lower T1 signal of normal brain and cerebrospinal fluid. *Early* subacute brain contusions contain intracellular methemoglobin and demonstrate very low signal on T2-weighted FLAIR (**G**), T2*-weighted series (SWI) (**H**), and T2-weighted series (**I**), whereas *late* subacute contusions contain extracellular methemoglobin and demonstrate high internal T2 signal (**B**) and a low-signal-intensity rim of hemosiderin on the T2*-weighted SWI series (**C**). FLAIR, fluid-attenuated inversion recovery; MRI, magnetic resonance imaging; SWI, susceptibility-weighted imaging.

Tables 1–3 summarize ratings by each pair of readers for brain contusion and TAI/DAI. We found very high interrater agreement for brain contusion, with kappa values for pairs of radiologists ranging from 0.84 to 0.87, PABAK from 0.92 to 0.94, PPA from 0.87 to 0.89, and NPA of 0.98 (Table 1). Figure 1 shows example cases of unanimous as well as partial agreement for brain contusion across the three readers.

**Table 1A.** Contusion on Brain MRI: Neuroradiologist 1 vs. Neuroradiologist 2

		Neuroradiologist 2		
		No	Yes	Totals
Neuroradiologist 1	No	434	10	444
	Yes	8	65	73
**Totals**		442	75	**517**

**Table 1B.** Contusion on Brain MRI: Neuroradiologist 1 vs. Neuroradiologist 3


		Neuroradiologist 3		
		No	Yes	Totals
Neuroradiologist 2	No	435	9	444
	Yes	7	66	73
**Totals**		442	75	**517**

**Table 1C.** Contusion on Brain MRI: Neuroradiologist 2 vs. Neuroradiologist 3

		Neuroradiologist 3		
		No	Yes	Totals
Neuroradiologist 1	No	432	10	442
	Yes	10	65	75
**Totals**		442	75	**517**

**Table 1D.** Contusion on Brain MRI: Kappa Statistic

	Neuroradiologist 1 vs. Neuroradiologist 2	Neuroradiologist 1 vs. Neuroradiologist 3	Neuroradiologist 2 vs. Neuroradiologist 3
Kappa	0.86 [0.79–0.92]	0.87 [0.81–0.93]	0.84 [0.78–0.91]
Prevalence and bias-adjusted kappa	0.93 [0.89–0.96]	0.94 [0.90–0.96]	0.92 [0.88–0.95]

**Table 1E.** Contusion on Brain MRI: Percent Agreement

	Neuroradiologist 1 vs. Neuroradiologist 2	Neuroradiologist 1 vs. Neuroradiologist 3	Neuroradiologist 2 vs. Neuroradiologist 3
Positive percent agreement	0.88 [0.81–0.93]	0.89 [0.83–0.94]	0.87 [0.80–0.92]
Negative percent agreement	0.98 [0.97–0.99]	0.98 [0.97–0.99]	0.98 [0.97–0.99]
Overall percent agreement	0.97 [0.95–0.98]	0.97 [0.95–0.98]	0.96 [0.94–0.97]

MRI, magnetic resonance imaging.

**FIG. 2.**
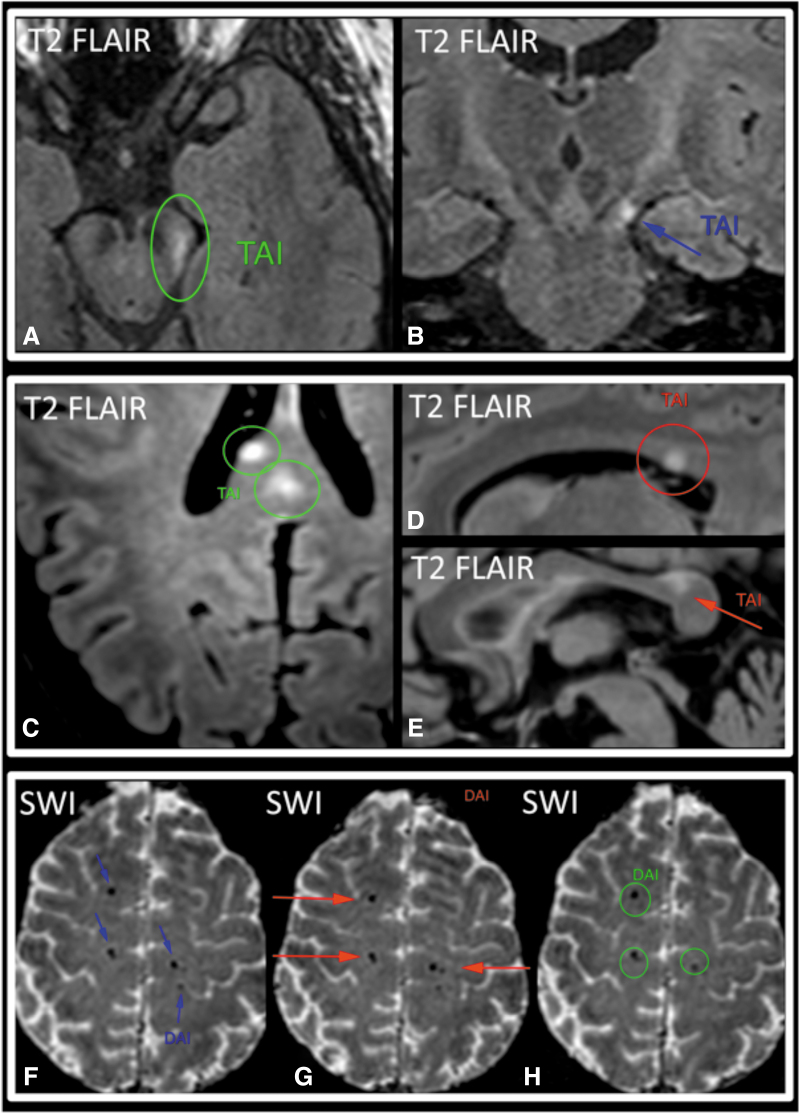
Traumatic axonal injury (TAI) and diffuse axonal injury (DAI). Two examples of partial (**A,B, C–E**) and one example of unanimous agreement (**F–H**) across three neuroradiologists. “Partial agreement” refers to MRI exams for which the three readers' initial interpretations regarding presence of TAI/DAI on the exam were not unanimous. Annotations from independent readers are shown in different colors (red, green, and blue) superimposed on the same image for demonstration purposes. (**A,B**) Single focus of T2-weighted FLAIR hyperintensity in the left cerebral peduncle (without susceptibility artifact), consistent with TAI. (**C–E**) Two foci of T2-weighted FLAIR hyperintensity in the body and splenium of the corpus callosum (without susceptibility artifact), also consistent with TAI. (**F–H**) Multiple (more than four) foci of susceptibility artifact in more than one lobe of the brain on T2*-weighted series (SWI), consistent with DAI. Additional foci marked by readers on other slices are not shown. FLAIR, fluid-attenuated inversion recovery; MRI, magnetic resonance imaging; SWI, susceptibility-weighted imaging.

TAI/DAI demonstrated intermediate kappa values. For TAI and DAI as elements of an ordinal scale (normal, TAI, and DAI), weighted kappa values for pairs of radiologists ranged from 0.65 to 0.72, and PABAK ranged from 0.74 to 0.78 (Table 2). Kappa values between pairs of radiologists for DAI ranged from 0.68 to 0.79, PABAK from 0.86 to 0.90, PPA from 0.72 to 0.82, and NPA from 0.96 to 0.97 (Table 3). Figure 2 shows examples of unanimous and partial agreement for TAI/DAI across the three readers.

**Table 2A.** TAI and DAI on Brain MRI: Neuroradiologist 1 vs. Neuroradiologist 2

		Neuroradiologist 2			
		None	TAI	DAI	Totals
Neuroradiologist 1	None	351	16	8	375
	TAI	35	32	7	74
	DAI	6	4	58	68
**Totals**		392	52	73	**517**

**Table 2B.** TAI and DAI on Brain MRI: Neuroradiologist 1 vs. Neuroradiologist 3

		Neuroradiologist 3			
		None	TAI	DAI	Totals
Neuroradiologist 1	None	353	19	3	375
	TAI	40	31	3	74
	DAI	12	12	44	68
**Totals**		405	62	50	**517**

**Table 2C.** TAI and DAI on Brain MRI: Neuroradiologist 2 vs. Neuroradiologist 3

		Neuroradiologist 3			
		None	TAI	DAI	Totals
Neuroradiologist 2	None	368	19	5	392
	TAI	25	26	1	52
	DAI	12	17	44	73
**Totals**		405	62	50	**517**

**Table 2D.** TAI and DAI on Brain MRI: Kappa Statistic

	Neuroradiologist 1 vs. Neuroradiologist 2	Neuroradiologist 1 vs. Neuroradiologist 3	Neuroradiologist 2 vs. Neuroradiologist 3
Kappa with linear weighting [95% CI]	0.72 [0.66–0.78]	0.65 [0.58–0.72]	0.67 [0.60–0.74]
Prevalence and bias-adjusted kappa	0.78 [0.74–0.82]	0.74 [0.70–0.78]	0.77 [0.73–0.81]

TAI, traumatic axonal injury; DAI, diffuse axonal injury; MRI, magnetic resonance imaging; CI, confidence interval.

**Table 3A.** Presence vs. Absence of DAI on Brain MRI: Neuroradiologist 1 vs. Neuroradiologist 2

		Neuroradiologist 2		
		No	Yes	Totals
Neuroradiologist 1	No	434	15	449
	Yes	10	58	68
**Totals**		444	73	**517**

**Table 3B.** Presence vs. Absence of DAI on Brain MRI: Neuroradiologist 1 vs. Neuroradiologist 3

		Neuroradiologist 3		
		No	Yes	Totals
Neuroradiologist 2	No	443	6	449
	Yes	24	44	68
**Totals**		467	50	**517**

**Table 3C.** Presence vs. Absence of DAI on Brain MRI: Neuroradiologist 2 vs. Neuroradiologist 3


		Neuroradiologist 3		
		No	Yes	Totals
Neuroradiologist 1	No	438	6	444
	Yes	29	44	1
**Totals**		467	50	**517**

**Table 3D.** Presence vs. Absence of DAI on Brain MRI: Kappa Statistic


	Neuroradiologist 1 vs. Neuroradiologist 2	Neuroradiologist 1 vs. Neuroradiologist 3	Neuroradiologist 2 vs. Neuroradiologist 3
Kappa [95% CI]	0.79 [0.72–0.87]	0.71 [0.62–0.81]	0.68 [0.58–0.78]
Prevalence and bias-adjusted kappa	0.90 [0.86–0.94]	0.88 [0.84–0.92]	0.86 [0.81–0.90]

**Table 3E.** Presence vs. Absence of DAI on Brain MRI: Percent Agreement

	Neuroradiologist 1 vs. Neuroradiologist 2	Neuroradiologist 1 vs. Neuroradiologist 3	Neuroradiologist 2 vs. Neuroradiologist 3
Positive percent agreement	0.82 [0.75–0.88]	0.75 [0.65–0.82]	0.72 [0.62–0.80]
Negative percent agreement	0.97 [0.96–0.98]	0.97 [0.95–0.98]	0.96 [0.95–0.97]
Overall percent agreement	0.95 [0.93–0.97]	0.94 [0.92–0.96]	0.93 [0.91–0.95]

DAI, diffuse axonal injury; MRI, magnetic resonance imaging; CI, confidence interval

### Consensus review

#### Contusion

All 61 cases initially identified as containing contusion by three readers, all 13 of 13 cases initially identified by two readers, and most (9 of 14) cases initially identified by only one reader were agreed to contain a contusion at consensus review. Thus, very few (only 5 of 27) cases of non-unanimous initial interpretation for presence of brain contusion were determined not to contain a true contusion upon consensus review.

#### Diffuse axonal injury

All 44 cases initially identified as containing DAI by three readers, 12 of 13 cases initially identified by two readers, but few (6 of 30) cases initially identified by only one reader were determined to contain DAI at consensus review. Thus, most (25 of 43) of the cases of non-unanimous initial interpretation for DAI were ultimately agreed not to contain DAI at consensus review.

#### Traumatic axonal injury

All 18 of 18 cases initially identified as containing TAI by three readers, 24 of 28 cases initially identified as TAI by two readers and normal by one reader, but only 16 of 43 cases initially identified as TAI by one reader and normal by two readers were determined to contain TAI at consensus review. Thus, many (31 of 71) cases of non-unanimous initial interpretation for TAI were agreed not to contain TAI or DAI on consensus review.

After consensus review, the final per-exam prevalence of brain contusion was 16% (83 of 517 exams). For TAI, the consensus results were: one lesion on 9% (45 of 517) of exams, two lesions on 4% (20 of 517), three lesions on 2% (8 of 517), and at least four lesions confined to one lobe of the brain on 0.4% (2 of 517) of exams. The consensus per-exam prevalence of DAI (at least four lesions including lesions in more than one lobe) was 12% (62 of 517 exams). Thus, the per-exam prevalences of brain contusion (16%) and TAI (15.4%) slightly exceeded the prevalence of DAI (12%) in this mTBI population.

## Discussion

In 2010, the NIH-NINDS published the first version of the TBI Imaging CDEs, consisting of standardized definitions of lesions in TBI,^9,10^ to satisfy “a need for a pathoanatomically based classification system for TBI if we are to successfully translate targeted therapies from the bench to the bedside.” This imaging-based classification scheme could be used throughout clinical research to enhance data quality and supplement the GCS, which, it was realized, was too crude an index of neurological injury severity, particularly for mTBI.

We found very high interrater agreement for presence of subacute brain contusion (PABAK from 0.92 to 0.94) on 2-week MRI. This is likely attributable to the high conspicuity and distinctive appearance of these lesions on T1-weighted images. Specifically, brain contusions (Fig. 1) generally demonstrate very high T1 signal in the 2-week subacute time frame, such that even tiny contusions well under 1 cm in size are often very prominent against the background low-to-intermediate T1 signal of the brain and cerebrospinal fluid. Subacute brain contusions are also often evident on T2, T2-weighted FLAIR, and/or T2*-weighted series, based on their T2 hypointensity in the early subacute time frame, T2 hyperintensity in the late subacute time frame, and peripheral rim of low signal on T2*-weighted images.

We found intermediate agreement for presence of TAI/DAI (PABAK from 0.74 to 0.78). The lower interrater agreement for TAI/DAI, compared to brain contusion, is likely because the most common manifestations of TAI/DAI on structural MRI overlap in appearance with other common findings that are unrelated to trauma. TAI/DAI on brain MRI are often identified as small round or linear areas of hemorrhage, which manifest as areas of signal void on T2*-weighted series. However, signal void in normal small arteries and veins, as well as artifactual foci of signal void, can mimic these tiny hemorrhages and thereby reduce interrater agreement. T2*-weighted series are highly prone to motion artifact, which also likely reduces interrater agreement.^16^ Another manifestation of TAI/DAI on structural MRI consists of T2-hyperintense foci (Fig. 2). Although some T2-hyperintense lesions can be confidently classified as TAI/DAI when they occur in locations that are characteristic of TAI/DAI (e.g., corpus callosum, cerebral peduncle), many others cannot be distinguished from the commonplace T2-hyperintense lesions of chronic small-vessel ischemic disease.

To place these results in context, a previous study reported a Fleiss kappa of 0.24 for interrater agreement in the detection of traumatic microhemorrhages on 3 Tesla brain MRI at 28 ± 3 weeks post-injury.^17^ To our knowledge, interrater agreement studies for detection of brain contusion on MRI have not been published. Regarding CT, Huff and colleagues^18^ reported a Fleiss kappa of 0.355 for three experienced neuroradiologists who classified 137 head CT exams into three categories (subarachnoid hemorrhage and/or brain contusion; subdural, epidural, and/or intracerebral hematoma; and normal, DAI, and/or cerebral swelling) in a multi-center study of acute mild-moderate TBI (GCS 9–15). In a study of interrater reliability among neuroradiologists and trauma neurosurgeons interpreting 50 consecutive head CT exams performed at their own level 1 trauma center, Chun and colleagues^19^ reported kappa values of 0.56–0.59 for any acute intracranial abnormality, 0.50–0.71 for subarachnoid hemorrhage, 0.21–0.63 for brain contusion, 0.42–0.73 for subdural hematoma, and 0.30–0.47 for intracerebral hematoma.

Discordance in interpretations can be attributable to a failure to perceive (detect) an abnormality (“I didn't see it”) and/or a difference in the interpretation of a perceived abnormality. Most (22 of 27) cases of non-unanimous initial interpretation for the presence of brain contusion were found, upon consensus review, to contain a true contusion and were likely attributable to failure to detect a small lesion. In contrast, most (25 of 43) cases of non-unanimous initial interpretation for presence of DAI were agreed upon at consensus review not to contain DAI and were thus attributable, at least partly, to an initial discordance in the interpretation of perceived finding(s); in these cases, signal void in small arteries and veins, as well as artifactual foci of signal void, likely played a role given that these were often difficult to definitively differentiate from punctate round/linear areas of slight hemorrhage that are a common hallmark of TAI/DAI on structural MRI.

### Strengths and limitations

We report the interrater reliability of the NIH-NINDS TBI Imaging CDEs, brain contusion, and TAI/DAI on MRI performed 2 weeks after mTBI. A strength is that this was a multi-center study that included scans using different MRI vendors and performed at different institutions, with interpretation by neuroradiologists trained at and practicing at different institutions. Also, in keeping with the intent of the NIH-NINDS CDEs to create standardized data-acquisition protocols whenever possible, a standardized MRI protocol was used across institutions. A limitation is that interrater agreement measured under conditions of a research investigation such as ours may differ from that in actual clinical practice. Optimization of image quality by tailoring protocols at each institution could have improved interrater agreement for the CDEs. In addition, familiarity with the appearance of MRI scans at one's own institution is likely to increase the accuracy of interpretations, particularly for CDEs such as TAI/DAI that may be mimicked by technical artifacts.

Clinical information, such as mechanism of injury, GCS score, and patient disposition (ED discharge, ward/intensive care unit [ICU] admission), and access to previous imaging, such as head CT exams, may improve raters' diagnostic accuracy and interrater agreement in clinical practice. Finally, brain MRI at an acute time point (<48 h post-injury) could demonstrate additional pathology, such as foci of reduced diffusion associated with TAI/DAI, but would have been logistically difficult to collect in large numbers, given that 1 week of lead time was typically needed to schedule outpatient MRI scans at many participating institutions.

We also note that our mTBI study population is enriched with patients on the more severe end of the mTBI spectrum: the initial head CT was positive for acute intracranial injury in 33%, whereas the average rate was only 9% for mTBI patients evaluated in U.S. EDs in 2009–2010.^1^ The higher rate of positive CT would be expected to carry over into higher prevalences of CDE findings on brain MRI. However, studies of interrater variability often use study populations that are enriched for findings of interest over a typical screening population, in order to limit the overall test set size to a reasonable number.^20^

In summary, the NIH-NINDS TBI imaging CDEs were created to enhance data quality and interoperability across sites and agencies over time. Measurement of interrater reliability is important for biomarker qualification by the FDA, which requests proof that an intended biomarker can be measured reliably. We found very high interrater agreement for brain contusion and intermediate agreement for TAI/DAI, both of which have been shown to have prognostic value in mTBI.^12^ Validated prognostic markers that identify mTBI patients at risk for unfavorable outcome may be useful for prioritizing patients for TBI-specific education and systematic follow-up, as well as for risk stratification of patients in clinical trials.
